# *Lactobacillus casei *strain GG in the treatment of infants with acute watery diarrhea: A randomized, double-blind, placebo controlled clinical trial [ISRCTN67363048]

**DOI:** 10.1186/1471-2431-4-18

**Published:** 2004-09-02

**Authors:** Eduardo Salazar-Lindo, Percy Miranda-Langschwager, Miguel Campos-Sanchez, Elsa Chea-Woo, R Bradley Sack

**Affiliations:** 1 Department of Pediatrics, Cayetano Heredia Hospital, Lima, Peru; 2 Department of Physics and Mathematics, Cayetano Heredia University, Lima, Peru; 3 Johns Hopkins Bloomberg School of Public Health, Department of International Health, Baltimore, Maryland, USA

## Abstract

**Background:**

Adjuvant therapy to ORT with probiotic bacteria for infants with acute watery diarrhea has been under active investigation. Most studies have been done in the developed world showing benefit only for viral mild gastroenteritis. We evaluated the effect of a milk formula containing one billion (10^9^) cfu/ml of *Lactobacillus casei *strain GG (LGG) upon duration and severity of diarrhea in infants in an environment with more severe acute diarrhea, where etiologic agents other than rotavirus are involved more frequently, and where mixed infections are more prevalent.

**Methods:**

Male infants aged 3–36 months brought for treatment of acute watery diarrhea of less than 48 hours were eligible. After rehydration was completed with the WHO's oral rehydration solution, patients were randomly assigned to receive a milk formula either containing LGG or not. Stool volume was periodically measured using a devise suited to collect stools separate from urine. Duration of diarrhea was estimated based on stools physical characteristics.

**Results:**

Eighty nine patients received the placebo milk formula and ninety received the LGG containing formula. Both groups were comparable in their baseline characteristics. Total stool output was significantly larger (p = 0.047) in the LGG group (247.8 ml/kg) than in the placebo group (195.0 ml/kg). No significant differences were found in duration of diarrhea (58.5 hours with LGG vs. 50.4 hours with placebo), rate of treatment failure (21.1% with LGG vs. 18.0% with placebo), and proportion of patients with unresolved diarrhea after 120 hours (12.2% with LGG vs. 12.5% with placebo). The rate of stools with reducing substances after 24 hours of treatment increased significantly in both groups (from 41.4% to 72.2% with LGG and from 45.9% to 68.0% with placebo).

**Conclusion:**

This study did not show a positive effect of LGG on the clinical course of acute watery diarrhea. Positive beneficial effects of LGG, as had been reported elsewhere, could have been masked in our study by worsening diarrhea due to transient lactose malabsorption. Further studies with low-lactose or non-lactose conveyors of LGG are desirable.

## Background

Diarrheal diseases in the developing world continue to cause significant morbidity and mortality, especially when associated with malnutrition [1]. Dehydration, potassium depletion and acidosis are the main life-threatening complications of the acute losses during watery diarrhea. More than 25 years of extensive use has demonstrated that these complications could be effectively treated or prevented by oral rehydration therapy [2]. However, the oral glucose-electrolyte rehydration solution such as that recommended by WHO and Unicef neither shortens the duration of the illness nor reduces the stool loss and may cause an increase in stool volume at least during the first hours in children with acute diarrhea [3]. Optimization of the standard WHO-ORS solution, by reducing its osmolarity, has been shown to reduce diarrhea duration, total stool output, and the need for unscheduled intravenous therapy [4].

Adjuvant therapy to ORT, based on oral administration of live probiotic bacteria aimed to improve recovery of infants from acute watery diarrhea, has been under active investigation [5]. These studies, done in the developed world, have shown a benefit only for viral mild gastroenteritis. Isolauri et al have shown that oral bacterial therapy with *Lactobacillus casei *strain GG (LGG) reduces both the severity and duration of acute non dehydrating rotavirus enteritis [6]. Another trial in Karelia republic, LGG was shown to decrease the duration of acute diarrhea in children with viral acute diarrhea but not in those with bacterial diarrhea [7]. In a multicenter study carried out in Europe, LGG was administrated in a hypotonic oral rehydration solution to children with acute diarrhea, showing that it was safe and resulted in shorter duration of diarrhea, less chance of a protracted course, and faster discharge from the hospital [8].

Limited information is available on the potential role of LGG in infants living in the developing world, with more severe acute watery diarrhea [9], where etiologic agents other than rotavirus are involved more frequently and where mixed infections are more prevalent [10]. We conducted this study aimed to assess the effect of a milk formula containing *Lactobacillus casei *strain GG (LGG) on the duration and severity of diarrhea in children with acute watery diarrhea from our hospital. The study was conducted in the Rehydration Unit of Cayetano Heredia Hospital in Lima, Peru from September 1991 to June 1992. The study protocol and Consent Form were reviewed and approved by the Ethical Committee of Cayetano Heredia University in compliance with the Helsinki Declaration.

## Methods

### Study population

Male infants 3–36 months of age brought for treatment because of acute diarrhea to the Rehydration Unit of Cayetano Heredia Hospital, Lima during September 1991 to June 1992 were eligible for the study if they met the following criteria: (a) a history of three or more watery stools per day for less than 48 hours, (b) no bloody stools at the moment of first examination, (c) clinical signs of dehydration, (d) no clinical features of hypovolemic shock, (e) no clinical signs of a coexisting acute systemic illness (i.e., meningitis, sepsis, pneumonia) or a recognized chronic disease such as pulmonary tuberculosis, and (f) no history of current antibiotic or antidiarrheal medication use. Patients 3–6 months old were considered for the study only if they were not on exclusive breastfeeding. Patients were not selected if their weight for age was less than 60% of the median weight for age established by the National Center for Health and Statistics (NCHS) [11]. Only patients whose parent or legal guardian was willing and able to give written informed consent were admitted to the study. Only male patients were selected in order to facilitate separate collection of stools and urine. The study size was calculated expecting a 33% reduction in duration of diarrhea and mean volume of diarrhea stool for a significance level of 0.05 and a power of 80%.

### Study design

Infants fulfilling the admission criteria were randomly assigned to a double-blind comparison of a milk formula containing 10^9 ^cfu/ml of *Lactobacillus casei *strain GG (LGG) or a similar milk formula not containing LGG. Both formulas were of identical looking and tasting. Both milk formulas were provided by the manufacturer (Valio Ltd, Helsinki, Finland) as milk powder to reconstitute with water. They were given in bottles, appropriately labeled and number-coded for each child to secure the double-blind design. The actual assignment of children to their study numbers was made at the time of their enrollment into the study. Each child admitted into the study was allocated to either treatment group by restricted randomization using random permuted blocks. The codes were prepared in Finland and kept in sealed envelopes in Lima.

### Baseline assessment

A clinical history and physical examination was completed upon admission to identify the patient, to determine the duration, type and severity of his diarrhea, to assess associated clinical features (fever, vomiting, dehydration, abdominal pain, distension), and to establish the nutritional status and previous dietary regimen of the subject. Blood for determination of serum sodium, potassium, chloride, glucose and bicarbonate, blood urea nitrogen, microhematocrit and plasma specific gravity was drawn from each patient before starting treatment. A routine urine analysis was also made. Fecal samples or rectal swabs taken at admission were transported in Cary-Blair transport medium to Cayetano Heredia University Microbiology Laboratory for primary isolation and identification of bacterial enteropathogens. A separate vial containing phosphate buffer saline was used to transport fecal specimens for rotavirus examination.

### Microbiologic methods

Specimens were cultured for *Salmonella*, *Shigella*, *E. coli *and Vibrios by standard direct and enrichment enteric media. *Campylobacter *was selectively cultured on sheep blood agar containing Butzler's antibiotic supplement and *Aeromonas *on ampicillin blood agar preceded by overnight enrichment in alkaline peptone water. Five lactose fermenting colonies of *E. coli *isolates per patient were serotyped according to standard methods using polyvalent sera for the recognition of enteropathogenic *E. coli*. Detection of Rotavirus was made by ELISA method using Rotazime^®^. Identification of enterotoxigenic strains of E. coli was not done. Details of the microbiologic methods used in this study are described in detail elsewhere [10].

### Rehydration

The degree of dehydration was assessed clinically. Dehydration was corrected and then fluid balance maintained using the oral rehydration salts (ORS) in its standard WHO's recommended formulation, following the WHO Guidelines [12]. Briefly, each child was given approximately 100 ml/kg of ORS during the first four hours. The ORS was administered in frequent sips using a spoon or by nasogastric tubes if vomit or stool output rates were high. Children were not given other fluid or foods during this period. Upon completion of this period, on-going fecal losses were replaced with the same solution, on a volume to volume basis until diarrhea ceased.

### Administration of the study formula

Once dehydration was corrected, feeding, including administration of the assigned study formula was initiated. The first dose of the assigned study formula was administered as soon as rehydration was completed and subsequent doses were given every four hours until cessation of diarrhea or for a maximum of five days. The formula was prepared immediately before administration of each dose by a specially trained nurse that was exclusively working for the study. Measuring spoons provided by the manufacturer were used and the formula powder was kept refrigerated all the time once the container was opened. Before use, the sachets with the study formulas were kept in a -20°C refrigerator. The formula powder was diluted in warm boiled water as to provide 670 Kcal per liter. The amount of formula given to each child was calculated based on the child's body weight, each child being given 150 ml/kg/day to a maximum of 1000 ml/day. On average, each serving of 100 ml will supply 10^11 ^cfu of LGG for those receiving the formula containing LGG. Administration of the formula was by bottle and not compulsive. If the child refused to drink the formula after reasonable attempts, no extraordinary methods, like delivery via nasogastric tubing, were used; in this situation the formula was offered again four hours later.

### Other foods given

Patients under six months of age were fed only the randomly assigned study formula to provide a daily caloric intake of at least 100 Kcal/kg (in a volume of 150 ml/kg/day). No other foods were given for these under six months patients. Older patients were fed the assigned formula *ad libitum *for a maximum of 1000 ml/day. Caloric requirements were completed with a blended soft baby food prepared from fresh ingredients (chicken breast, rice, carrots, squash, potatoes, and vegetable oil), by the Hospital's Nutrition Department. This soft baby food provides 1 Kcal per gram (approximately, 10% of its calories as protein, 45% as carbohydrates and 45% as fats). A total of at least 100 Kcal/kg/day (including the calories provided by the study formula) were offered to these children.

### Measurement of clinical outcomes

All oral intake (ORS, study formula, breast milk, plain water, soft baby food) as well as urine, stool and vomitus volume were strictly measured and recorded during hospitalization. For this purpose patients were put on metabolic beds (which have a hole in the mattress were a container was placed to allow direct stool collection). A urine collection bag was fitted to avoid contamination of stools with urine and to measure the urine volume. After the first 72 hours, differential weight of dry and soiled diapers was used instead the metabolic bed to determine stool weight if the previous observed purging rate was below 5 ml/kg/hour. Blood analyses taken at admission were repeated at 24 hours. Stool examination for pH, glucose, reducing substances, leukocytes and microscopic fat were performed at admission, 24, 72 hours and at discharge. Urine specific gravity was determined every 24 hours until discharge.

### Criteria for stopping treatment and discharging the patient

Patients were discharged from the study 24 hours after cessation of diarrhea, or at the end of five days from admission, or at the time treatment failure occurred. At discharge each patient was categorized as have completed the trial, as a treatment failure, as an unresolved diarrhea situation, or as a withdrawal.

### Primary outcomes

• Rate of treatment failure: proportion of patients in each study group who have recurrence or continued presence of more than 5% dehydration, worsening electrolyte abnormalities, no weight gain since admission, developing of ileus or severe diarrhea defined as a purging rate in excess of 10 ml/Kg/hr in two consecutive 4-hour periods.

• Rate of unresolved diarrhea after five days of treatment: proportion of patients in each study group with continuing diarrhea after five days of treatment.

• Duration of diarrhea: time in hours from admission until cessation of diarrhea

• Duration of hospitalization: time in hours from admission until discharge from hospital. Patients with treatment failure are excluded.

• Total stool output: volume of diarrheic stools collected from admission until cessation of diarrhea or for a maximum of 120 hours if diarrhea continues, expressed in milliliters per kilogram of body weight.

• Total intake of oral rehydration solution: volume of ORS taken from admission until cessation of diarrhea or for a maximum of 120 hours if diarrhea continues, expressed in milliliters per kilogram of body weight.

### Secondary outcomes

• Total study formula intake: volume of study formula taken from admission until cessation of diarrhea or for a maximum of 120 hours if diarrhea continues, expressed in milliliters per kilogram of body weight.

• Total energy intake: energy from all sources (ORS, formula) taken from admission until cessation of diarrhea or for a maximum of 120 hours if diarrhea continues, expressed in kilocalories per kilogram of body weight.

• Total volume of vomitus: volume of vomitus collected each day from admission until cessation of vomitus or for a maximum of 120 hours, expressed in milliliters per kilogram of body weight.

• Total volume of urine: volume of urine collected each day from admission until cessation of diarrhea or for a maximum of 120 hours, expressed in milliliters per kilogram of body weight.

### Working definitions

The following definitions were used for this study:

• Carbohydrate malabsorption: stool sample giving a positive reaction for reducing substances (0.75 g % or above).

• Cessation of diarrhea: passage of formed stool or passage of no stool for 12 consecutive hours.

• Early withdrawal: patient who (a) developed a complicating illness, (b) presented bloody diarrhea after admission and before five days of treatment, (c) was prematurely discharged under request of his parent or legal guardian, or (d) had any protocol violation or non-compliance.

### Statistical analysis

The data, initially collected in pre-coded forms, were entered in a database organized with a relational model under Fox Pro^® ^v. 1.02 (Dbase^® ^compatible formats) using a data entry program with on-line checking. The data was checked again in batch mode and finally transferred to EpiInfo^® ^for Windows^® ^v. 3.01 (November 2003) where the tabulations and statistical analysis were performed. Letters (A or B, maintaining blindly the treatment group assignment) identified treatment groups until all statistical analysis was completed. Only after completing the analysis investigators unblinded the treatment assignment. Baseline and outcome two-way comparisons were made between the two treatment groups. A 5% level of significance for statistical tests was set in advance for all comparisons. Chi-square Yates corrected was used to compare discrete variables. One way analysis of variance was performed for continuous variables. Data from patients prematurely withdrawn from the study was included in the analysis up to the time of withdrawal (intention-to-treat analysis).

## Results

**Figure 1:**
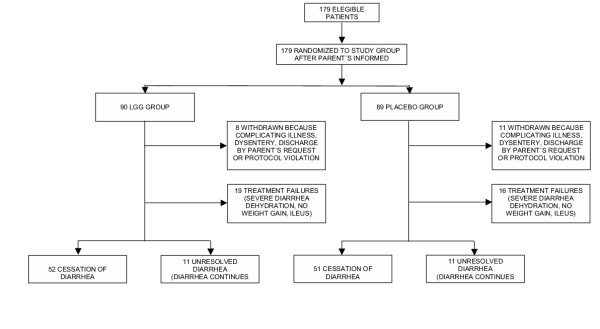
Diagram showing the flow of study subjects throughout the study

A total of 179 patients were admitted into the study, 90 in the LGG group and 89 in the control group. Nineteen patients, eight from the LGG group and 11 from the control group, were prematurely withdrawn from the study (figure 1) because of either one of the following reasons: bloody stools within the first 24 hours after admission (11 patients); parental non-compliance (four patients); no diarrheal stools passed within the first 24 hours since admission (two patients); typical severe cholera-like diarrheal disease improperly included (one patient); and severe systemic infection present but not recognized at admission (one patient).

### Baseline characteristics

Table 1 presents the baseline characteristics of patients upon admission. The treatment groups were comparable in age, nutritional status, and other clinical and laboratory variables. Marginal differences were found, however, in stool output during the first four hours of the rehydration phase, before administration of the study formula. The LGG group was thus comprised by slightly more severe cases. Table 2 shows stool microbiology and parasitology at admission. The distribution of enteropathogens was similar between both groups, except for rotavirus which was found more frequently in the placebo group (39.3%) than in the LGG group (24.4%) with a marginal statistical significance for the difference (p = 0.052).

**Table 1:** Clinical and laboratory features on admission

	**Placebo group**	**N**	**LGG group**	**N**	**P**
Age (months)	14.7 ± 6.4	89	14.9 ± 7.5	90	0.878
Duration of diarrhea before admission (h)	29.5 ± 14.9	89	28.8 ± 14.9	90	0.733
N° of stool motions 24 h before admission	6.6 ± 3.2	89	7.5 ± 3.9	90	0.101
N° of patients with vomitus 24 h before admission	64 (71.9%)	89	66 (73.3%)	90	0.963
N° of patients with fever 24 h before admission	39 (43.8%)	89	43 (47.8%)	90	0.703
Clinical dehydration		89		90	0.165
Mild	48 (53.9%)		36 (40.0%)		
Moderate	40 (44.9%)		52 (57.8%)		
Severe	1 (1.1%)		2 (2.2%)		
% of weight gain after rehydration	3.0 ± 2.2	89	3.1 ± 2.3	90	0.763
Fever at admission (≥ 37.5°C)	16 (18.0%)	89	14 (15.9%)	88	0.868
Weight for height*	94.8 ± 9.3	89	94.1 ± 7.6	90	0.551
Height for age*	88.9 ± 12.1	89	97.2 ± 3.7	90	0.546
Weight for age*	88.9 ± 12.1	89	88.9 ± 10.8	90	0.952
Stool output (ml/kg) first 4 h (rehydration phase)	14.4 ± 12.4	89	18.1 ± 16.1	90	0.085
Hematocrit (%)	35.0 ± 4.2	89	35.5 ± 3.8	90	0.426
Plasma specific gravity	1.028 ± 0.002	89	1.028 ± 0.002	90	0.769
Serum Na^+ ^(mEq/l)	136.6 ± 4.7	89	136.4 ± 4.6	87	0.770
Serum K^+ ^(mEq/l)	4.0 ± 0.7	89	4.0 ± 0.7	87	0.985
Serum Cl (mEq/l)	102.5 ± 5.2	89	102.6 ± 5.4	87	0.874
Serum total CO2 (mmol/l)	14.0 ± 3.9	88	14.0 ± 3.9	87	0.971
Blood urea nitrogen (mg/dl)	13.1 ± 10.5	70	11.4 ± 6.6	73	0.253
Blood glucose (mg/dl)	107.5 ± 33.4	88	106.5 ± 35.9	88	0.848
N° of patients with fecal leukocytes (> 20 PMN/hpf)	30 (34.5%)	87	21 (23.6%)	89	0.154
N° of patients with microscopic fat	56 (64.4%)	87	52 (58.4%)	89	0.513
Fecal pH	6.3 ± 1.1	87	6.6 ± 1.1	89	0.126
N° of patients with CHO malabsorption	39 (45.9%)	85	36 (41.4%)	87	0.659

* % of NCHS median

Values are mean ± SD or n (%)

**Table 2:** Stool microbiology and parasitology at admission

	**Placebo group**	**N**	**LGG group**	**N**	**P**
Rotavirus	33 (39.3%)	84	22 (24.4%)	90	0.052
EPEC	12 (17.6%)	68	9 (13.2%)	68	0.635
*Vibrio cholerae*	7 (8.0%)	88	11 (12.4%)	89	0.471
*Campylobacter sp*.	6 (6.8%)	88	9 (10.1%)	89	0.605
*Shigella sp*.	9 (10.2%)	88	4 (4.5%)	89	0.241
*Aeromonas sp*.	2 (2.3%)	88	1 (1.1%)	89	0.992
*Salmonella sp*.	0 (0.0%)	88	1 (1.1%)	89	0.995
Negative microbiology	25 (36.8%)	68	32 (47.1%)	68	0.452
*Cryptosporidium parvum*	6 (7.0%)	86	2 (2.2%)	89	0.256
*Giardia lambliae*	2 (2.3%)	86	3 (3.4%)	89	0.969
*Strongyloides stercolaris*	1 (1.2%)	86	0 (0.0%)	89	0.986
*Trichomonas sp*.	0 (0.0%)	86	2 (2.2%)	89	0.492
Negative parasitology	77 (89.5%)	86	82 (92.1%)	89	0.738
Negative microbiology and parasitology	22 (35.5%)	62	30 (44.8%)	67	0.371

No patients were found positive for the following parasites: Ascaris, Enterobius, Trichuris, *E. hystolytica*, Ancylostoma, Necator.

ETEC identification was not done

Values are n (%)

### Stool purging reduction

The mean total stool output (Table 3) was lower in the placebo group (195.0 ml/kg) than in the LGG group (247.8 ml/kg) with a marginal statistical significance for the difference (p = 0.047). The difference between means in total stool output was -52.8 ml/kg, with 95% confidence limits between -105.4 to -0.2 ml/kg.

**Table 3:** Clinical outcomes

	**Placebo group**	**N**	**LGG group**	**N**	**P**	**Difference between means**
Discharge condition		89		90		
Early withdrawal	11 (12.4%)		8 (8.9%)		0.609	
Treatment failure	16 (18.0%)		19 (21.1%)		0.734	
Cessation of diarrhea	51 (57.3%)		52 (57.8%)		0.931	
Unresolved diarrhea	11 (12.45%)		11 (12.2%)		0.842	
Mean duration of diarrhea (hours)*	50.4 ± 28.0	51	58.5 ± 30.2	52	0.157	-19.6 to 3.4
Mean duration of hospitalization (hours)**	74.7 ± 33.7	62	81.2 ± 32.6	63	0.280	-18.4 to 5.4
Total stool output (ml/kg)	195.0 ± 171.9	89	247.8 ± 180.2	90	0.047	-105.4 to -0.2
Total oral rehydration solution intake (ml/kg)	236.5 ± 195.6	89	272.8 ± 188.1	90	0.208	-93.7 to 21.1
Total study formula intake (ml/kg)	215.5 ± 136.3	89	231.5 ± 136.6	90	0.435	-56.8 to 24.8
Total energy intake (Kcal/kg)^§^	220.7 ± 140.4	89	247.4 ± 143.0	90	0.209	-69.1 to 15.7
Total volume of vomitus (ml/kg)	27.6 ± 45.5	89	27.4 ± 33.9	90	0.968	-11.8 to 12.2
Total volume of urine (ml/kg)	81.5 ± 65.2	89	87.1 ± 69.4	90	0.579	-25.7 to 14.5

* For patients who ceased diarrhea before 120 hours.

** Patients with treatment failure or early withdrawals are excluded.

^§ ^Calculated from calories provided by study formula, soft foods, breastmilk, and oral rehydration solution.

Values are mean ± SD, n (%), or 95% confidence limits.

### Duration of diarrhea

The proportion of patients with unresolved diarrhea after 120 hours was similar in both treatment groups (table 3). For those patients that ceased diarrhea during the study period the mean duration of diarrhea was similar between groups (table 3). The difference between means in duration of diarrhea was 8.1 hours with 95% confidence limits between -19.6 hours to 3.4.

### Blood chemistry

No patient had to be withdrawn from the study because of worsening blood chemistry abnormalities.

### Carbohydrate malabsorption

The proportion of patients with carbohydrate malabsorption 24 hours after admission into the study was high although not different between the treatment groups (table 4). However, the rate of carbohydrate malabsorption after 24 hours of treatment increased significantly in both treatment groups with respect to admission (table 4). The pH of stools at 24 hours after admission is also significantly lower in both groups compared to the pH at admission, a result consistent with worsening carbohydrate malabsorption (table 4).

**Table 4:** Comparison of stool characteristics at admission and 24 hours after

	**Admission**	**N**	**24 h later**	**N**	**P**
Fecal pH					
Placebo group	6.3 ± 1.1	87	5.9 ± 0.8	76	0.014
LGG group	6.6 ± 1.1	89	6.1 ± 0.9	74	0.004
CHO malabsorption					
Placebo group	39 (45.9%)	85	51 (68.0%)	75	0.008
LGG group	36 (41.4%)	87	52 (72.2%)	72	< 0.001

Values are mean ± SD or n (%)

No adverse effects due to the study formula were notice in either group during the study.

## Discussion

Children included into this study probably represent the type of patients with diarrheal disease most frequently seen at a health service in Peru before the cholera epidemic of 1991. They were infants under two years of age, with an episode of acute watery diarrhea, presenting with signs of mild to moderate dehydration and a slight compromise of the nutritional status. Thus, we can conclude that the results of this study can be applied to most cases of acute watery non-cholera diarrhea seen in this population setting.

The comparison of baseline characteristics showed no significant differences between groups. Marginal differences (not statistically significant) were observed in two variables that measure severity of illness: stool frequency 24 hours before admission and stool output during rehydration phase. Both were higher in the LGG group. Other indicators of severity, like clinical estimate of dehydration, percentage of weight gain after rehydration and proportion of patients with vomitus before admission, were similar between both groups. On the other hand, there were more cases (not statistically significant) of rotavirus in the placebo group than in the LGG group. In order to make sure that the groups were in fact comparable, we perform the statistical adjustment for these variables using multiple regression analysis and found no association with the principal outcomes (results of this analysis are not shown in the report).

In more than 55% of patients an enteropathogen could be identified. This figure could be higher because studies for ETEC were not done. The most prevalent agents were rotavirus (30.7%), EPEC (11.7%), *Vibrio cholerae *(10.1%) and *Campylobacter sp*. (8.4%), with an important proportion of patients having mixed infections. These findings differ considerably from those of the Finnish clinical trial in which LGG was also tested for efficacy in reducing acute infantile diarrhea [6]. In the Finnish trial, rotavirus accounted for more than 80% of diarrheal cases, with no cases of bacterial diarrhea. A setting of greater diversity of etiologic agents and frequently co-pathogen association is perhaps more representative of developing countries [10].

The first topic to be discussed here is the high proportion of patients that did not recovered optimally during the trial, a condition that did not depend on the assigned treatment group. About 12% of patients had an unresolved diarrhea and an additional 20% were classified as treatment failures, mostly due to severe diarrhea. In two clinical trials conducted at our unit similar rates were found. For instance, in a clinical trial designed to test the clinical efficacy of a citrate-based ORS in children with acute watery diarrhea, patients who received standard therapy (WHO-ORS) had a treatment failure rate of about 24% [13]. This rate was 40% if only rotavirus associated diarrhea was considered. In another study, in which a chicken soup-based ORS was evaluated for children with similar characteristics (not published), 15.4% treatment failure rate for all subjects standard and 20% for those with rotavirus was observed. In the Finnish study, all children recovered before 5 days and no treatment failures were reported [6]. This striking difference could be explained in part by dissimilar etiologies as was mentioned above. Bacterial diarrhea and infection with more than one pathogen might produce a more severe illness. The nutritional status is also important to explain the difference. It has been well established from studies in developing countries that diarrhea lasts longer in poorly nourished children [14]. This difference seems to be important even when only slight malnutrition is present, as is the case for the patients admitted into this study.

Except for total stool output (which showed a marginal statistical significance), no significant differences in the principal outcomes between groups were found. Duration of diarrhea, duration of hospitalization, and total ORS intake were not significantly different between the experimental and placebo group. The mean volume of formula (and of lactose) administered to children was the same for both groups. The confidence limits of the difference between the two groups indicate that a maximum reduction on duration of diarrhea of 6.7% attributable to the LGG formula could have been detected with the sample size of this study. These numbers indicate that the study does have a sample size sufficient to reject a type II error. No positive effects of treatment could be demonstrated as opposed to Isolauri's experience in Finland [6]. The dose factor could not explain the difference between the two studies. The dose of LGG in Isolauri's study was 2 × 10^10–11 ^cfu per day [6]. The dose of LGG in our study was 6–8 × 10^11 ^cfu per day, well above the threshold dose of 10 billion cfu suggested as most effective [15]. Viability of the *Lactobacillus GG *in the sachets while stored in Lima was not confirmed bacteriologically.

There were slightly fewer cases of rotavirus in the LGG group than in the placebo group (24.4% vs. 39.3%), although the difference was not statistically significant. LGG appears to be more effective in viral than in bacterial diarrhea [8]. These facts could explain in part the lack of beneficial effect of LGG in our study. It is possible that LGG was not able to colonize the gut of our patients in the presence of bacterial pathogens. Although colonization has been documented during rotavirus diarrhea in children [16], to our knowledge this ability has not been demonstrated when other intestinal pathogens are involved. Colonization properties of LGG in patients with bacterial enteritis would deserve further studies.

Our study clearly shows that the rate of carbohydrate malabsorption raised in both treatment groups from 43.6% at admission to 70.1% 24 hours later. The amount of lactose offered to patients in this study was much higher than in the usual feeding regimen at our unit (10.5 g/kg/day vs. 3.7 g/kg/day, respectively). Although to some investigators lactose intolerance is becoming a decreasing problem [17], it is clear from these data that lactose malabsorption as a factor cannot be dismissed. We had consistently found a high proportion of patients with carbohydrate malabsorption in all similar studies conducted in our unit. Thus, one possible explanation for the lack of efficacy of LGG in our patients is that worsening diarrhea due to lactose malabsorption could have masked any beneficial effect of the LGG. If this is true, then reducing the lactose content of the formula might reveal a positive effect. Another option is to deliver the LGG in suitable non-lactose containing infant food that could be given to diarrheic children as part of their treatment. Carefully conducted studies might prove this hypothesis.

## Conclusions

Both groups were comparable in their baseline characteristics. Marginal differences were found in variables that measured severity of illness before treatment. Total stool output was slightly higher in the LGG group reaching a marginal statistical significance. No significant differences in the other principal outcome variables were found between treatment groups. The proportion of patients with unresolved diarrhea after 120 hours was similar in both treatment groups. For those patients that ceased diarrhea during the study period the mean duration of diarrhea was similar between groups. The rate of carbohydrate malabsorption after 24 hours of treatment increased significantly in both treatment groups.

## Competing interests

None declared.

## Authors' contributions

ES-L participated in the design of the study, organized and coordinated the study, participated in the statistical analysis and drafted the manuscript. PM-L carried out the clinical work, participated in the statistical analysis and the drafting of the manuscript. MC-S carried out the statistical analysis. EC-W participated in the clinical work. RBS conceived of the study, and participated in its design and drafting of the manuscript. All authors read and approved the final manuscript

## Pre-publication history

The pre-publication history for this paper can be accessed here:


